# Validation of an abbreviated Treatment Satisfaction Questionnaire for Medication (TSQM-9) among patients on antihypertensive medications

**DOI:** 10.1186/1477-7525-7-36

**Published:** 2009-04-27

**Authors:** Murtuza Bharmal, Krista Payne, Mark J Atkinson, Marie-Pierre Desrosiers, Donald E Morisky, Eric Gemmen

**Affiliations:** 1 Quintiles Inc, Falls Church, Virginia, USA; 2 United BioSource Corporation, Montreal, Canada; 3 University of California, San Diego, California, USA; 4 UCLA School of Public Health, Los Angeles, California, USA

## Abstract

**Background:**

The 14-item Treatment Satisfaction Questionnaire for Medication (TSQM) Version 1.4 is a reliable and valid instrument to assess patients' satisfaction with medication, providing scores on four scales – side effects, effectiveness, convenience and global satisfaction. In naturalistic studies, administering the TSQM with the side effects domain could provoke the physician to assess the presence or absence of adverse events in a way that is clinically atypical, carrying the potential to interfere with routine medical care. As a result, an abbreviated 9-item TSQM (TSQM-9), derived from the TSQM Version 1.4 but without the five items of the side effects domain was created. In this study, an interactive voice response system (IVRS)-administered TSQM-9 was psychometrically evaluated among patients taking antihypertensive medication.

**Methods:**

A total of 3,387 subjects were invited to participate in the study from an online panel who self-reported taking a prescribed antihypertensive medication. The subjects were asked to complete the IVRS-administered TSQM-9 at the start of the study, along with the modified Morisky scale, and again within 7 to 14 days. Standard psychometric analyses were conducted; including Cronbach's alpha, intraclass correlation coefficients, structural equation modeling, Spearman correlation coefficients and analysis of covariance (ANCOVA).

**Results:**

A total of 396 subjects completed all the study procedures. Approximately 50% subjects were male with a good racial/ethnic mix: 58.3% white, 18.9% black, 17.7% Hispanic and 5.1% either Asian or other. There was evidence of construct validity of the TSQM-9 based on the structural equation modeling findings of the observed data fitting the Decisional Balance Model of Treatment Satisfaction even without the side effects domain. TSQM-9 domains had high internal consistency as evident from Cronbach's alpha values of 0.84 and greater. TSQM-9 domains also demonstrated good test-retest reliability with high intraclass correlation coefficients exceeding 0.70. As expected, the TSQM-9 domains were able to differentiate between individuals who were low, medium and high compliers of medication, with moderate to high effect sizes. There was evidence of convergent validity with significant correlations with the medication adherence scale.

**Conclusion:**

The IVRS-administered TSQM-9 was found to be a reliable and valid measure to assess treatment satisfaction in naturalistic study designs, in which there is potential that the administration of the side effects domain of the TSQM would interfere with routine clinical care.

## Background

Patient satisfaction with their medication is shown to affect treatment-related behaviors, such as their likelihood of continuing to use their medication, to use their medication correctly and to adhere with medication regimens [1-7]. Although a number of disease-specific measures of patients' treatment satisfaction (TS) and treatment satisfaction with their medication (TS-M) have been reported in the literature [8-18], very few studies have attempted to assess a more general measure of TS-M that would permit comparisons across medication types and patient conditions. The Treatment Satisfaction Questionnaire for Medication (TSQM) is a widely used generic measure to assess TS-M and has been psychometrically validated in a heterogeneous sample [19,20].

The development of the TSQM along with the conceptual framework of TS and patients' satisfaction with their medication has been described in detail earlier [19,20]. In the development of the TSQM, an initial set of 55 items were drafted to represent the conceptual framework of TS-M identified based on qualitative research which included the concepts of effectiveness, symptom relief, side effects, convenience, tolerability, impact on daily life and functioning and global satisfaction [19]. After item refinement and psychometric validation, the TSQM Version 1.4 is comprised of 14 questions that provide scores on four scales: effectiveness (3 items), side effects (5 items), convenience (3 items) and global satisfaction (3 items) [19].

Based on the conceptual framework of TS-M, patients' perception of side effects with their medication is an important component of satisfaction with their medication. However, the use of the TSQM with the side effects domain has a potential to interfere with real-world outcomes which are central to naturalistic study designs. For example, a recent study in patients treated with antiepileptic drugs found that a significant higher rate of adverse event reporting occurred among patients who were administered a checklist versus those reporting them spontaneously. The study also found that reporting of adverse events resulted in changing treatment administered [21]. The study findings demonstrate the potential of a questionnaire like the TSQM with its side effect domain to interfere with naturalistic studies which are designed to collect data from the usual clinical practice environment with minimum interference to the behaviors of study participants (both patients and physicians).

In the real world, physicians must collect and report suspected adverse events to medications already on the market according to established guidelines for adverse event reporting and their own professional discretion. Thus, in a naturalistic study of the usual care of hypertension management, the administration of a questionnaire, such as the TSQM, which queries the patient about their experience in relation to side effects, has a potential to provoke the physician to assess the presence or absence of adverse events in a way that is not typical for clinical practice, as demonstrated in the study by Carreño and colleagues [21]. This artificial trigger for adverse event questioning has the potential to impact naturalistic study outcomes – particularly those that relate to care patterns, treatment satisfaction and medication compliance [21].

This study discusses the psychometric validation of the TSQM-9, which uses nine of the 14 TSQM Version 1.4 items not including five TSQM questions (ie, questions 4 to 8) related to side effects of medication. The TSQM-9 has been developed to provide a suitable measure of treatment satisfaction with medication in such naturalistic studies where measuring patient-reported side effects has a potential to interfere with the study objectives. The objective of this study was to psychometrically validate the interactive voice response system (IVRS)-administered abbreviated 9-item TSQM (TSQM-9) in a sample of patients taking hypertensive medications.

## Methods

### Study sample

Study subjects were recruited from an online population of patients, reporting to be hypertensive, identified by Synovate Healthcare (Chicago, Illinois, USA). Synovate has recruited a large number of U.S. subjects to participate in surveys of different healthcare related topics. These subjects, considered healthcare panelists, must consent and be 18 years of age or older to participate.

This study was approved by an independent ethics committee. The study recruited subjects with a goal of achieving at least 300 completed subjects as an accepted sample size for validation studies [22].

### Study design

Out of the 25,600 healthcare panelists that met the inclusion criteria for the study, a random sample of 3,387 subjects were sent an email invitation in which a web link directed them to the TSQM-9 study enrollment website, within which the study rationale, objectives and procedures were fully described. To participate in the TSQM-9 validation study, subjects confirmed in this website that they had read the description of the study design and required procedures and they wished to continue with the enrollment process. If so, the subject opted-in via a web link, which was considered as an informed consent. Upon receipt of the 'opt-in' response, subjects were automatically directed to a confirmation of study eligibility web page, where they answered a few brief questions confirming study eligibility and provided their primary contact telephone number. The eligibility questions included confirmation on whether they had hypertension and whether they were taking prescription medications for their hypertension.

Once eligibility was confirmed via the website, the subject was sent a confirmation email that provided a reiteration of study procedures, a toll-free telephone number and a unique randomized access code which enabled secure access to the telephone-based interactive voice response system (IVRS) within which the study questions were implemented. Subjects were invited to call the IVRS as soon as possible (preferably the same day as study enrollment). Each study subject was instructed to call the IVRS and enter study data twice: the first assessment (time 1) and a second assessment within 7 to 14 days (time 2). During the first call, subjects completed the TSQM-9 and the modified Morisky Scale questions [23]. In the second assessment only the TSQM-9 questions were completed.

### Study measures

#### Abbreviated Treatment Satisfaction Questionnaire for Medication (TSQM-9)

The TSQM Version 1.4 is a 14-item psychometrically robust and validated instrument consisting of four scales [19]. The 14 questions were selected from an original set of 55 questions obtained from literature review and focus groups. The four scales of the TSQM include the effectiveness scale (questions 1 to 3), the side effects scale (questions 4 to 8), the convenience scale (questions 9 to 11) and the global satisfaction scale (questions 12 to 14). In the TSQM-9, the five items related to side effects of medication were not included, which creates a need to psychometrically assess the performance of the abbreviated instrument.

The TSQM-9 domain scores were calculated as recommended by the instrument authors, which is described in detail elsewhere [19,20]. The TSQM-9 domain scores range from 0 to 100 with higher scores representing higher satisfaction on that domain.

#### Modified Morisky scale

The modified Morisky scale is a 7-item instrument to assess self-reported patient adherence modified from the validated 8-item Morisky scale developed to assess adherence related to antihypertensive medication [23]. Items of the 8-item Morisky scale are described in Table 1. Further description and psychometric data on the 8-item Morisky scale are described in detail elsewhere [23].

**Table 1:** Items of the Modified Morisky Scale

Items	Response format
Do you sometimes forget to take your [health concern] pills?	Yes or No
	
People sometimes miss taking their medications for reasons other than forgetting. Thinking over the past two weeks, were there any days when you did not take your [health concern] medicine?	Yes or No
	
When you travel or leave home, do you sometimes forget to bring along your [health concern] medication?	Yes or No
	
Did you take your [health concern] medicine yesterday?	Yes or No
	
When you feel like your [health concern] is under control, do you sometimes stop taking your medicine?	Yes or No
	
Taking medication everyday is a real inconvenience for some people. Do you ever feel hassled about sticking to your [health concern] treatment plan?	Yes or No
	
How often do you have difficulty remembering to take all your medications?	Never/Rarely, Once in a while, Sometimes, Usually, All the time

All translations, adaptations, computer programs, and scoring algorithms, and any other related documents of the Morisky Medication Adherence Scale (MMAS 4- and 8-item versions), are owned and copyrighted by, and the intellectual property of, Donald E. Morisky, ScD, ScM, MSPH. Professor of Community Health Sciences, UCLA School of Public Health, Los Angeles, CA 90095-1772.

One item of the 8-item Morisky scale related to stopping medication because of feeling worse with the medication ('Have you ever cut back or stopped taking your medication without telling your doctor, because you felt worse when you took it?' was not included in the modified Morisky scale due to similar concerns about the item interfering with the treatment process in a naturalistic study design. Based on communication with the author of the Morisky scale, deleting this item resulted in only a very minor change in the internal consistency of the scale from 0.83 to 0.82. Sensitivity and specificity of the 7-item modified Morisky scale for identifying lower vs. higher adherers was 91% and 50%, respectively, which was close to the estimates reported for the original 8-item scale at 93% and 53%, respectively.

The modified Morisky scale yield a total score with a range of 0 to 7, with higher scores indicating higher adherence to medication. The scores of the modified Morisky scale can be categorized as low compliers (< 6), medium compliers (> = 6 but <7) and high compliers (= 7) based on its criterion validity with blood pressure control.

### Statistical methods

The construct validity of the TSQM-9 was evaluated using structural equation modeling (SEM) based on the factor structure outlined by the Decisional Balance Model of Treatment Satisfaction used by Atkinson et al. (2005) for the TSQM [20]. Briefly, based on the Decisional Balance Model of Treatment Satisfaction, dimensions of treatment experience (effectiveness, convenience and side effects) are weighted by individuals to predict global satisfaction and subsequent treatment adherence. In the TSQM-9, since the side effect domain of the TSQM is not included, it becomes important to assess the construct validity of the TSQM-9 using the Decisional Balance Model of Treatment Satisfaction, with respect to the ability of its scales to predict treatment satisfaction even without the side effect domain.

Structural equation modeling (SEM) helps to model the hypothetical relationships between observed and latent variables. The measurement and structural model to be tested is pre-specified by defining the relationships among the variables (ie, items) and latent constructs (ie, scales), and then tested by examining the fit between the specified model and the correlation or covariance patterns that are observed in the data. If the proposed model fits the observed data, it is said to be confirmed. The fit of the specified model was evaluated by reviewing two criteria, the global fit measures including the Bentler's comparative fit index (CFI), the Bentler and Bonett's non-normed fit index (NNFI) and chi-square, and the magnitude of the individual standardized parameter estimates for the paths in the model. To demonstrate a good model fit, the chi-square test should be non-significant, and the CFI and NNFI should be close to or exceeding 0.90 [24,25]. The magnitude of the individual standardized parameter estimates for the paths in the model should be statistically significant and ideally be greater than or equal to 0.70 [24].

Internal consistency of the three scales of the TSQM-9 (ie, effectiveness, convenience and global satisfaction) was assessed using Cronbach's alpha at time 1 and time 2 [26]. Test-retest reliability of the TSQM-9 was assessed using the intraclass correlation coefficient using data from the two time periods (time 1 and time 2) that were separated by 7 to 14 days. Assuming that there is no significant change in the factors that affect patient satisfaction with medication during the short time interval in the two administrations of the TSQM-9, patient responses from the two time periods were expected to have a high correlation.

Known-group validity analysis was conducted to determine the ability of the TSQM-9 to discriminate among patients known to differ in their satisfaction with medication. It is expected that individuals that are more compliant are likely to be more satisfied with their medication. TSQM-9 domain scores at time 1 were compared between low compliers (modified Morisky scale score<6) and medium compliers (modified Morisky scale score > = 6 but <7) using analysis of covariance (ANCOVA) controlling for covariates which were significantly related to treatment satisfaction in bivariate analysis (patient age, gender and race/ethnicity). Since only one individual was classified as high complier (modified Morisky scale score = 7), this group was excluded from the known-group validity analysis.

Effect size based on Cohen's d (difference between the mean score of the groups/pooled standard deviation) were calculated to assess the magnitude of group differences [27]. An effect size of ≥ 0.50–<0.80 is considered as moderate while an effect size ≥ 0.80 is considered as large [28]. Convergent validity of the TSQM-9 was assessed by the correlation of the modified Morisky scale score and the TSQM-9 domain scores at time 1 using the Spearman rank-order correlation coefficients. As satisfaction with medication is expected to be positively associated with medication compliance, a moderate to high positive correlation (> = 0.25) between the scores was expected [19]. All analyses were conducted in SAS version 9.1 for Windows [29].

## Results

### Study subjects

**Figure 1:**
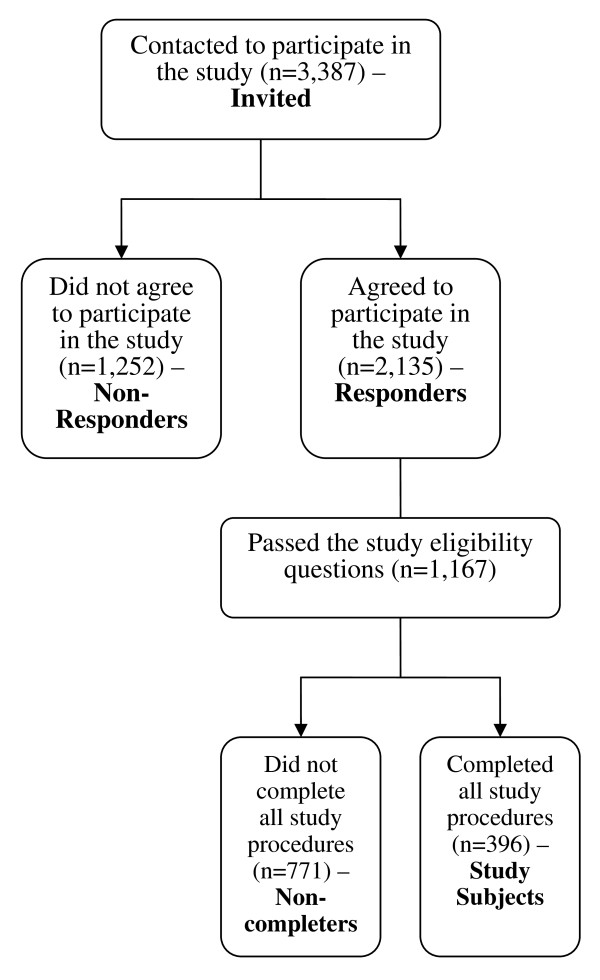
**Study Sample**.

A total of 2,135 subjects (63.0%) out of the 3,387 subjects that were contacted, agreed to participate in the study. A total 968 subjects (45.3%) out of the 2,135 responders were screen failures since they did not pass the study eligibility questions. Of the 1,167 that were enrolled in the study, a total of 396 subjects (33.9%) completed all the study procedures (required assessments at time 1 and time 2) and were used in the current analysis (see Figure 1).

The mean (standard deviation) age of the study subjects was 55.1 (11.4) years. Approximately 50% of the subjects were male. There was a good racial/ethnic mix among the subjects with 58.3% white, 18.9% black, 17.7% Hispanic and the rest belonging to Asian or other non-Hispanic category.

### Comparisons of responders versus non-responders

Significant differences were observed between responders to the study invitation (n = 2,135) and non-responders (n = 1,252) on age, gender and race/ethnicity. Responders were older (55.1 years versus 52.5 years; p < 0.0001), more likely to be male (50.2% versus 36.1%; p < 0.0001), more likely to be white (56.1% versus 45.9%) and less likely to be black (15.1% versus 20.1%) (p < 0.0001) compared to non-responders.

### Comparisons of study subjects versus non-completers

No significant differences were observed between study subjects (n = 396) and non-completers (n = 771) on gender and age. However, study subjects were less likely to be Hispanic (17.7% versus 23.7%) and more likely to be white (58.3% versus 51.0%) (p < 0.0001) compared to non-completers.

### Comparisons of study subjects versus subjects invited to participate in the study

No significant differences were observed between study subjects (n = 396) and individuals initially invited to participate in the study (n = 3,387) on gender and age. However, study subjects were less likely to be Hispanic (17.7% versus 25.6%) and more likely to be white (58.3% versus 52.3%) (p < 0.0001) compared to those invited to participate in the study.

### TSQM-9 observed scores

Table 2 describes the TSQM-9 domain scores at time 1 and time 2. TSQM scores have a range of 0 to 100, with higher scores indicating higher satisfaction. Similar scores were observed at time 1 and time 2 for all the TSQM-9 domains. Mean (SD) score on the effectiveness domain was 73.4 (18.5) at time 1 and 73.7 (17.3) at time 2. Mean (SD) score on the convenience domain was 78.7 (15.9) at time 1 and 79.3 (15.5) at time 2. Mean (SD) score on the global satisfaction domain was 75.5 (18.6) at time 1 and 76.6 (18.8) at time 2.

**Table 2:** Summary Scores on TSQM-9 Domains and Modified Morisky Scale and Test-Retest Reliability of the TSQM-9

Scale	Time 1	Time 2	Intraclass Correlation Coefficient, ICC (95% CI of ICC)
TSQM-9			
Effectiveness			0.784 (0.757, 0.811)
N	396	396	
Mean (SD)	73.44 (18.51)	73.74 (17.27)	
Median	72.22	72.22	
Minimum, maximum	5.56, 100	0, 100	
			
Convenience			0.737 (0.704, 0.768)
N	396	396	
Mean (SD)	78.69 (15.89)	79.32 (15.46)	
Median	83.33	83.33	
Minimum, maximum	16.67, 100	16.67, 100	
			
Global Satisfaction			0.759 (0.729, 0.788)
N	396	396	
Mean (SD)	75.52 (18.61)	76.57 (18.79)	
Median	78.57	78.57	
Minimum, maximum	0, 100	0, 100	
			
Modified Morisky Scale			
N	396	NA	
Mean (SD)	5.02 (1.27)		
Median	5.25		
Minimum, maximum	0.25, 7		

Time 1 = Day of first IVRS call after enrollment; Time 2 = Time 1 + 7 to 14 days.

Intraclass correlation coefficient (ICC) computed using TSQM-9 scores at Time 1 (Day of first IVRS call after enrollment) and Time 2 (Time 1 + 7 to 14 days)

Possible range on TSQM-9 domain scores is 0 to 100.

Possible range on modified Morisky Scale score is 0 to 7

### Modified Morisky scale observed scores

Table 2 also describes the modified Morisky scale score at time 1. Modified Morisky scale score has a range of 0 to 7, with higher scores indicating higher adherence to medication. The mean (SD) adherence to medication among the study subjects was at 5.0 (1.3).

### Construct validity of the TSQM-9

**Figure 2:**
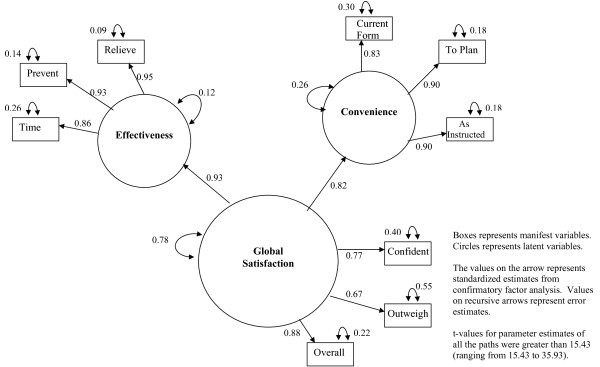
**Structural Equation Modeling Analysis for the TSQM-9**.

Figure 2 depicts a diagrammatic representation of the structural equation modeling analysis of the TSQM-9 based on the Decisional Balance Model of Treatment Satisfaction (without the side effects domain). The model tested included a measurement model, which described the relationship of the manifest variables that measure the latent constructs (Effectiveness, Convenience and Global Satisfaction), and a causal model, which described the relationship of the latent constructs with each other. For testing the above model using structural equation modeling (SEM), as recommended by Hatcher, the variances of the exogenous variables (latent constructs) need to be specified as free parameters to be estimated [24]. To solve the resulting scale indeterminancy issue caused by estimating the variances of the exogenous variables in the above model, one factor loading for each latent construct was fixed to 1 (Item # Relieve for Effectiveness; Item # To Plan for Convenience; Item # Overall for Global Satisfaction) [24]. The specified model was confirmed as overidentified with number of data points (information = 45) exceeding the number of parameters to be estimated (parameters estimated = 20) [24].

As seen in Figure 2, the model fit is acceptable for most of the criteria. Although the observed chi-square test was significant (Chi-square: 117.4; df = 25; Chi-square/df = 4.7; p-value < 0.0001), this test is regarded as being very sensitive to sample size, rendering it unclear in many situations whether the statistical significance of the chi-square statistic is due to poor fit of the model or to the size of the sample, warranting the need to use other indices to assess model fit [24]. Both, the CFI value of 0.9712 and NNFI value of 0.9585 exceeded 0.90, suggesting a model with adequate fit. The individual standardized parameter estimates for the paths in the model were high and most greater than 0.70. All the t-values for parameter estimates were greater than 15.43, far exceeding the critical value of 1.96 required for statistical significance at an alpha of 0.05. The model results indicated that independently 86.3% and 67.6% of the variance in global satisfaction is explained by effectiveness and convenience domains, respectively.

Based on these results, there is evidence to suggest that the observed data fits the specified Decisional Balance Model of Treatment Satisfaction (without the side effects domain), demonstrating construct validity of the TSQM-9, even without the side effects domain of the TSQM.

### Internal consistency of the TSQM-9

As described in Table 3, all the item-total correlations were greater than 0.65. All Cronbach's alpha values exceeded 0.80 at time 1 and time 2, demonstrating good internal consistency [30]. The Cronbach's alpha values at time 1 and time 2, respectively, were 0.94 and 0.92 for the effectiveness domain, 0.91 and 0.92 for the convenience domain, and 0.84 and 0.85 for the global satisfaction domain.

**Table 3:** Internal Consistency of the TSQM-9 Items

	Time 1		Time 2	
	
Items and Domains	Item-total correlation	Cronbach's Alpha	Item-total correlation	Cronbach's Alpha
Effectiveness		0.935		0.924
How satisfied or dissatisfied are you with the ability of the medication to prevent or treat your condition?	0.878		0.840	
How satisfied or dissatisfied are you with the way the medication relieves your symptoms?	0.904		0.892	
How satisfied or dissatisfied are you with the amount of time it takes the medication to start working?	0.819		0.805	
				
Convenience		0.911		0.915
How easy or difficult is it to use the medication in its current form?	0.791		0.799	
How easy or difficult is it to plan when you will use the medication each time?	0.841		0.854	
How convenient or inconvenient is it to take the medication as instructed?	0.834		0.833	
				
Global Satisfaction		0.837		0.848
Overall, how confident are you that taking this medication is a good thing for you?	0.755		0.768	
How certain are you that the good things about your medication outweigh the bad things?	0.684		0.698	
Taking all things into account, how satisfied or dissatisfied are you with this medication?	0.694		0.684	

Time 1 = Day of first IVRS call after enrollment; Time 2 = Time 1 + 7 to 14 days

### Test-retest reliability of the TSQM-9

Table 2 describes the test-retest reliability of each of the domains of the TSQM-9 using the intraclass correlation coefficient (ICC). As expected, the ICC values were high: 0.784 for the effectiveness domain, 0.737 for the convenience domain and 0.759 for the global satisfaction domain, demonstrating test-retest reliability of the TSQM-9. Given the 4-week recall period used in the TSQM-9, subjects completing TSQM-9 at time 1 and a second assessment within 7 to 14 days had a sufficient overlap in time period for assessing satisfaction between the two time periods, and thus not expected to have any bias in the test-retest reliability analysis.

### Known-group validity of the TSQM-9

Known-group validity analysis determines the ability of the TSQM-9 to discriminate among patients known to differ in their satisfaction with medication. Table 4 compares TSQM-9 domains among low, medium and high compliers at time 1. Since only one individual was classified as high complier (modified Morisky scale score = 7), this group was excluded from the known-group validity analysis. As expected, TSQM-9 domain scores were significantly different between the two groups, with higher scores (greater satisfaction) among medium compliers compared to low compliers. The analysis controlled for patient age, gender and race/ethnicity, which were found to be significantly related to treatment satisfaction in bivariate analysis. Adjusted mean scores (lsmean) on the effectiveness domain were 66.1 among low compliers and were significantly higher at 77.1 among medium compliers. Adjusted mean scores on the convenience domain were 71.7 among low compliers and were significantly higher at 84.0 among medium compliers. Adjusted mean scores on the global satisfaction domain were 68.4 among low compliers and were significantly higher at 79.3 among medium compliers. The effect size for the mean differences in the TSQM-9 domain scores was moderate to large and ranged from 0.65 to 0.88 when comparing medium compliers with low compliers [28].

**Table 4:** Known-group Validity of the TSQM-9

					Effect Size
					
Domain	Low Compliers (Modified Morisky Scale < 6)	Medium Compliers (Modified Morisky Scale > = 6 but < 7)	High Compliers (Modified Morisky Scale = 7)	p-value	Medium Compliers – Low Compliers
Effectiveness				< 0.0001	0.68
n	200	195	1		
Lsmean (SE)	66.08 (1.93)	77.11 (1.97)	NA		
Mean (SD)	67.53 (18.85)	79.37 (16.07)	100 (-)		
Median	66.67	83.33	100		
Minimum, maximum	5.56, 100	11.11, 100	100, 100		
					
Convenience				< 0.0001	0.88
n	200	195	1		
Lsmean (SE)	71.67 (1.60)	84.02 (1.64)	NA		
Mean (SD)	72.31 (16.42)	85.13 (12.31)	100 (-)		
Median	72.22	83.33	100		
Minimum, maximum	16.68, 100	50, 100	100, 100		
					
Global Satisfaction				< 0.0001	0.65
n	200	195	1		
Lsmean (SE)	68.36 (1.94)	79.27 (1.98)	NA		
Mean (SD)	69.82 (19.86)	81.25 (15.19)	100 (-)		
Median	71.43	85.71	100		
Minimum, maximum	0, 100	21.43, 100	100, 100		

p-value and lsmean for compliance level based on analysis of covariance (ANCOVA) model controlling for patient age, gender and race/ethnicity; Since only one individual in the study was classified as a high complier, analyses were conducted among low and medium compliers only.

Effect size based on Cohen's d;

### Convergent validity of the TSQM-9

Convergent validity of the TSQM-9 was assessed by correlation of the modified Morisky scale score and the TSQM-9 domain scores at time 1 using the Spearman rank-order correlation coefficient. As satisfaction with medication is expected to be positively associated with medication adherence, a moderate-to-high positive correlation between the scores is expected. TSQM-9 convenience domain had the largest correlation with the medication adherence score at 0.46, followed by effectiveness domain scores at 0.38 and global satisfaction at 0.34.

## Discussion

This study provides evidence of the reliability and validity of the IVRS-administered abbreviated 9-item TSQM without the side effects domain (TSQM-9). There was evidence of construct validity based on structural equation modeling findings of the observed data fitting the Decisional Balance Model of Treatment Satisfaction even without the side effects domain. TSQM-9 domains had high internal consistency as evident from Cronbach's alpha values of 0.84 and over. TSQM-9 domains also demonstrated good test-retest reliability, with high intraclass correlation coefficients exceeding 0.70. As expected, the TSQM-9 domains were able to differentiate between individuals who were medium and low compliers with a moderate effect size. There was also evidence of convergent validity, with significant correlations with the medication adherence scale.

The TSQM-9 was developed due to the need for using a measure of treatment satisfaction that was designated to minimize interference in routine clinical care in the context of naturalistic study designs. The side effect domain of the TSQM Version 1.4 queries the patient about their experience in relation to side effects and has a potential to provoke the physician to assess the presence or absence of adverse events in a way that is clinically atypical, affecting the naturalistic design of a study [21]. It should be noted that we do not recommend the use of TSQM-9 over the earlier versions of the TSQM in clinical studies where there is no such possibility of the side effects domains interfering with study objectives and where the outcome of investigational drugs are being studied. Clearly, based on the conceptual framework of TS-M, patient's perception of side effects with their medication is an important component of satisfaction with their medication. However, there are specialized studies in which the side effects domain has potential to interfere with objectives of the study; the TSQM-9 is intended to provide a validated instrument for such scenarios.

It is important to note that although the side effects domain was not included in TSQM-9, any unpleasant experiences with a medication are likely to be captured in the TSQM global satisfaction items. As a result, even without the side effects items, the TSQM-9 allows for patients to weigh the pros and cons of medication and the less favorable aspects of patients' experiences with their medications would be captured.

In this study, we found that the convenience domain had strongest association with medication adherence followed by effectiveness and global satisfaction. In previous TSQM validation analysis, global satisfaction had the strongest association with medication adherence [20]. This association may be reflective of the hypertensive patient population. In an asymptomatic chronic condition like hypertension, the convenience domain becomes an important factor for medication adherence given that the patient has to take their medications daily without any apparent symptomatic changes in their condition.

One of the limitations of this study was that it was conducted in a homogenous sample of patients using hypertensive medications. Since the TSQM is a generic measure of patients' satisfaction with their medication, validation in a more heterogeneous representative sample, containing, for example, patients with different chronic medical conditions would have improved the robustness of results. Future studies on the performance of the TSQM-9 in other patient populations are recommended. In this study, differences were observed on demographic characteristic between responders to the study invitation and non-responders. Further, there were some differences on race/ethnicity between study completers and non-completers. However, given that the purpose of this study was instrument validation and that the study subjects used in the analysis had a good gender and racial/ethnic mix, these differences are unlikely to bias the study results.

Another potential limitation of this study is the use of a 7-item modified Morisky scale for the validation of TSQM-9. An item from the original Morisky scale related to stopping medication because of feeling worse with the medication was dropped due to similar concerns about the item interfering with the treatment process in a naturalistic study design [21]. However, as discussed earlier, deleting this item resulted in minimal change in the internal consistency of scale as well as the sensitivity and specificity of the scale for identifying lower vs. higher adherers.

Despite these limitations, the TSQM-9 may prove to be a useful measure to assess treatment satisfaction with medication in patients with hypertension when real-world outcomes are of interest and there is a need to minimize interference to the behaviors of health care providers and patients alike.

## Conclusion

The IVRS-administered TSQM-9 was found to be a reliable and valid measure to assess treatment satisfaction in naturalistic study designs, when there is potential for the side effects domain of the TSQM to interfere with routine clinical care and the objectives of the study.

## Competing interests

The study was funded by Novartis Pharmaceuticals.

## Authors' contributions

MB: Psychometric Design and Analysis, Project Management, Primary Authorship

KP: Project Management, Study Design and Planning, Second Authorship

MA: Study Design and Planning, Contributing Author

MPD: Study Design and Planning, Contributing Author

DM: Study Design and Planning, Contributing Author

EG: Psychometric Design and Analysis, Project Management, Contributing Author
